# Fifteen-gene expression based model predicts the survival of clear cell renal cell carcinoma

**DOI:** 10.1097/MD.0000000000011839

**Published:** 2018-08-17

**Authors:** Ping Li, He Ren, Yan Zhang, Zhaoli Zhou

**Affiliations:** a Shanghai University of Medicine & Health Sciences; b School of Optical-electrical and Computer Engineer of University of Shanghai for Science and Technology; c Shanghai Key Laboratory for Molecular Imaging, Collaborative Research Center, Shanghai University of Medicine & Health Science; d Department of Pharmacology, School of Pharmacy, Shanghai University of Medicine & Health Science, Shanghai, China.

**Keywords:** ccRCC, expression, prognosis

## Abstract

Clear-cell renal cell carcinoma (ccRCC) is the major renal cell carcinoma subtype, but its postsurgical prognosis varies among individual patients.

We used gene expression, machine learning (random forest variable hunting), and Cox regression analysis to develop a risk score model based on 15 genes to predict survival of patients with ccRCC in the The Cancer Genome Atlas dataset (N = 533). We validated this model in another cohort, and analyzed correlations between risk score and other clinical indicators.

Patients in the high-risk group had significantly worse overall survival (OS) than did those in the low-risk group (*P* = 5.6e-16); recurrence-free survival showed a similar pattern. This result was reproducible in another dataset, E-MTAB-1980 (N = 101, *P* = .00029). We evaluated correlations between risk score and other clinical indicators. Risk was independent of age and sex, but was significantly associated with hemoglobin level, primary tumor size, and grade. Radiation therapy also had no effect on the prognostic value of the risk score. Cox multivariate regression showed risk score to be an important indicator for ccRCC prognosis. We plotted a nomogram for 3-year OS to facilitate use of risk score and other indicators.

The risk score model based on expression of the 15 selected genes can predict survival of patients with ccRCC.

## Abstract 2

Supplemental Digital Content is available in the text

## 1 Introduction

An estimated 66,800 new cases of renal cell carcinoma (RCC) and 23,400 RCC-related deaths occurred in China in 2015.^[1]^ Among RCC subtypes, more than 75% of diagnoses are of clear-cell renal cell carcinoma (ccRCC). However, predicting survival of patients with ccRCC is challenging because of its genetic heterogeneity.^[2]^ Biomarkers that can guide prognosis prediction and drug development for ccRCC are therefore needed.

Many biomarkers including mRNAs, long noncoding RNAs, miRNAs, and proteins have been widely reported to predict prognosis in ccRCC. For example, in ccRCC, overexpression of FABP7 reportedly promotes cell growth and predicts poor outcome,^[3]^ high RAB25 expression is associated with poor survival,^[4]^ and enhanced CX3CR1 expression promotes migration and proliferation.^[5]^ Some miRNAs have been associated with survival in ccRCC.^[6]^ Low miR-497 expression reportedly predicts poor survival in ccRCC patients.^[7]^ Long noncoding RNA *CADM1-AS1* was also shown to promote growth and migration.^[8]^ However, no single biomarker offers predictability across datasets, due to the genetic heterogeneity of ccRCC.

Models based on expression of multiple genes have been developed to predict survival of some cancers, and have been validated across datasets and study populations.^[6,9–12]^ Although models have been developed for ccRCC, their robustness and clinical usefulness are limited.

Here, by screening survival-related genes in The Cancer Genome Atlas (TCGA) dataset, in combination with random forest variable hunting and Cox multivariate regression, we have developed a prognostic model. Patients in the model's high-risk group had significantly worse survival than those in the low-risk group, and this finding was further validated in another dataset. We also analyzed correlations between risk score (RS) and clinicopathological indicators.

## 2 Material and methods

### 2.1 Data processing

This study does not involve new participants; thus an ethics committee or institutional review board approval is not necessary. Raw expression data for ccRCC in TCGA dataset were downloaded from the UCSC Xena (http://xena.ucsc.edu/public-hubs/) in a log2 (RSEM + 1) transformed format. The data were further transformed to log2 (RSEM) with R. Clinical information was also downloaded from the same website and manually curated.

Processed microarray data (E-MTAB-1980) was downloaded from the ArrayExpress (http://www.ebi.ac.uk/arrayexpress/) web site. The processing method has been previously described.^[13]^ Clinical indicators and follow-up information was further manually curated.

### 2.2 Cox univariate and multivariate regression

Cox univariate regression was implemented in TCGA dataset using R package “survival.” *P* values were calculated for each gene, and genes significantly associated with overall survival (OS; false discovery rate [FDR] <0.00001, adjusted with method “BH”) were retained as list 1. Using the median expression value of each gene as cut-off, samples were divided into gene-high and gene-low groups, and OS differences between these groups was evaluated; genes with FDR <0.0001 were selected as list 2. Genes presented in both list 1 and list 2 were retained for further analysis. Random forest variable hunting was implemented with these selected genes to optimize the gene panel, with 100 repeats and 100 iterations. Cox multivariate regression was performed to estimate RS with the 15 genes obtained in the previous step. The RS was calculated as 
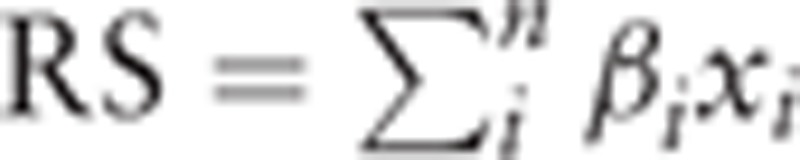
, where *β*_*i*_ refers to the coefficient of each gene calculated, and *x*_*i*_ indicates the relative expression value of corresponding gene.

### 2.3 Statistical analysis

All statistical analyses in this study were performed with R and R packages. The Cox probability hazard model was performed with R package “survival.” ROC curves were plotted with R package “pROC,”^[14]^ and “randomForestSRC” was used to perform random forest survival variable hunting. The nomogram was plotted with R package “rms.”

## 3 Results

### 3.1 Survival genes identification

**Figure 1:**
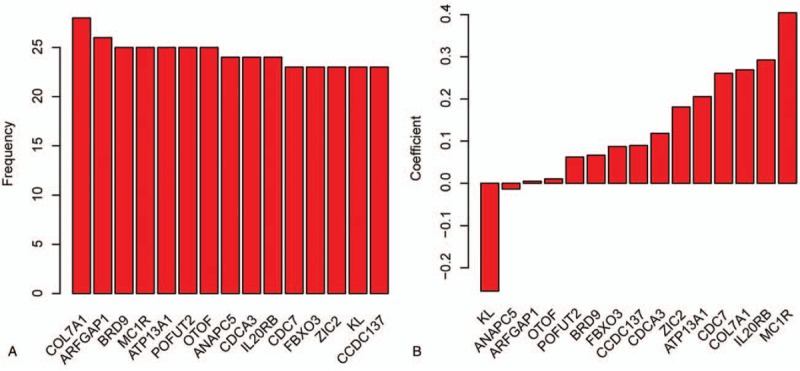
Genes selected for risk score model. (A) Gene frequency in variable hunting and (B) multivariate Cox regression coefficient for each gene.

Survival analyses were performed in TCGA dataset (N = 533). Cox univariate regression was used to correlate expression level of each gene with OS; genes significantly associated with survival (FDR < 0.00001) was retained for further analysis (termed as gene list 1). Samples in TCGA dataset were then divided into gene-high and gene-low groups according to the median expression level of each gene, and survival differences were compared between these 2 subgroups (termed as gene list 2). Survival-associated genes (FDR < 0.00001) were retained. Genes in both list 1 and list 2 were identified for further analysis, and 75 genes were identified. Random forest variable selection was carried out to optimize and narrow down the panel. Finally, 15 genes were identified (Fig. 1A, Table 1). The RS was calculated as: RS = (0.0896∗*CCDC137*) + (−0.2552∗*KL*) + (0.1807∗*ZIC2*) + (0.0869∗*FBXO3*) + (0.2608∗*CDC7*) + (0.2924∗*IL20RB*) + (0.1183∗*CDCA3*) + (−0.0137∗*ANAPC5*) + (0.0104∗*OTOF*) + (0.0620∗*POFUT2*) + (0.2056∗*ATP13A1*) + (0.4044∗*MC1R*) + (0.0664∗*BRD9*) + (0.0049∗*ARFGAP1*) + (0.2689∗*COL7A1*). The gene symbol indicates the relative expression level. Coefficients of each gene are shown in Fig. 1B. Positive coefficients suggest that the gene is negatively associated with survival time/rates; genes with negative coefficients are positively associated survival.

**Table 1:**
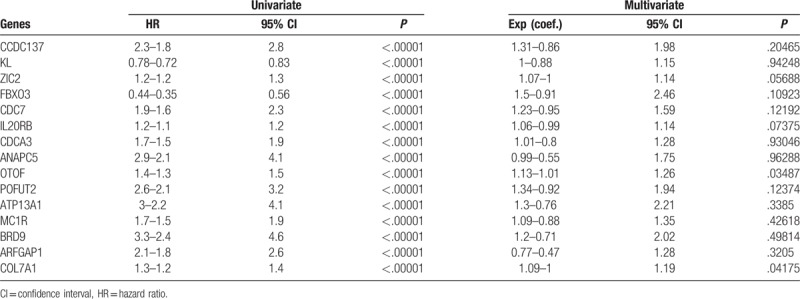
Coefficients of genes selected.

### 3.2 Risk score in TCGA dataset

**Figure 2:**
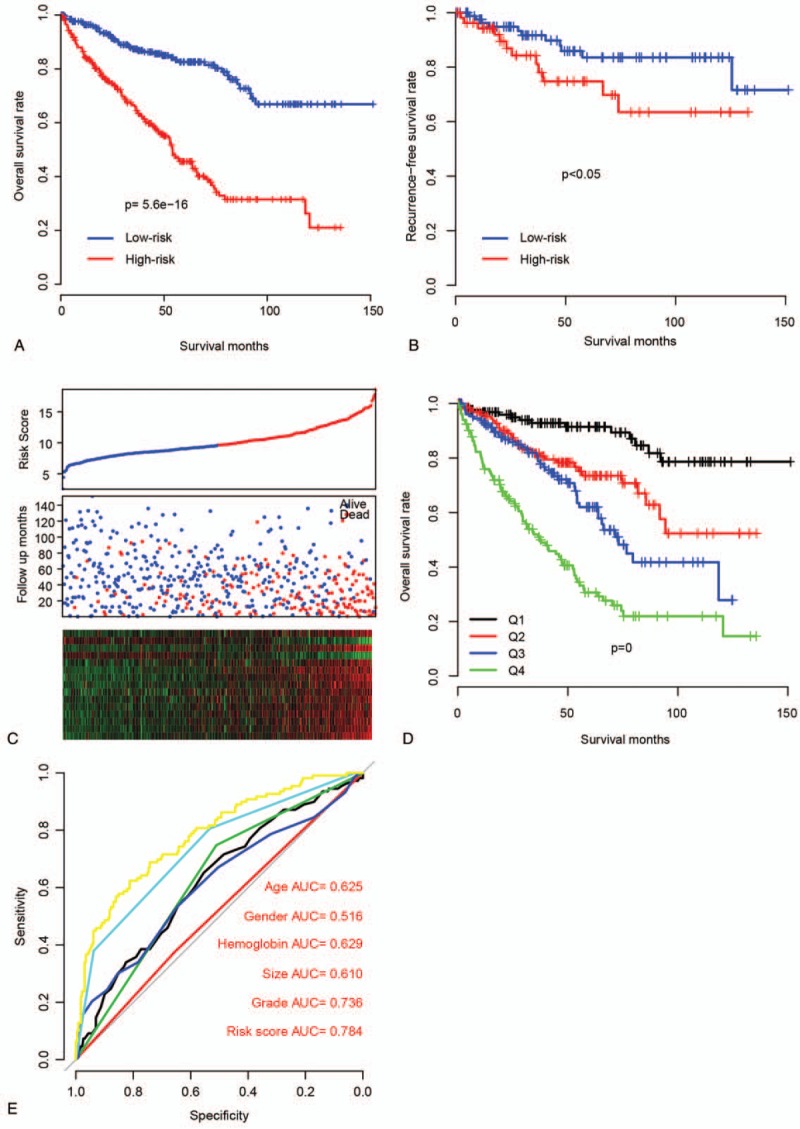
Prognostic effect of risk score on training dataset. (A) Overall survival and (B) recurrence-free survival differed between high-risk and low-risk groups. (C) Detailed survival information and expression patterns of candidate genes also differed between high-risk and low-risk groups (top: risk score; middle: survival status; bottom: candidate gene expression profiles). (D) Survival difference in quartiles was also compared. (E) Three-year survival by areas under the curve (AUCs) for risk score and other clinical information.

The performance of the RS was assayed in TCGA dataset. After calculating the RS of each patient using the aforementioned formula, the samples in TCGA dataset were divided into low-risk and high-risk groups according to the median RS in this dataset, and their survival differences were compared. Patients in low-risk group had a significantly better prognosis than the high-risk group (Fig. 2A, N = 533, *P* = 5.6e-16; detailed survival information is shown in Supplementary Table 1). Recurrence-free survival (RFS) in the 2 groups was also compared, and the result is consistent with the OS profile (Fig. 2B). In addition, we divided the samples in TCGA dataset into quartiles, and assayed the survival difference among subgroups (Fig. 2D), and similar results were seen. Patients with high RS usually had early events, and unique expression pattern of the 15 genes (Fig. 2C). We plotted areas under the curve (AUCs) for 3-year OS with respect to age (0.625), sex (0.516), hemoglobin (0.629), primary tumor size (0.610), grade (0.736), and RS (0.784; Fig. 2E). Collectively, these results indicate that RS can help predict survival of patients with ccRCC.

### 3.3 Risk score in validate dataset

**Figure 3:**
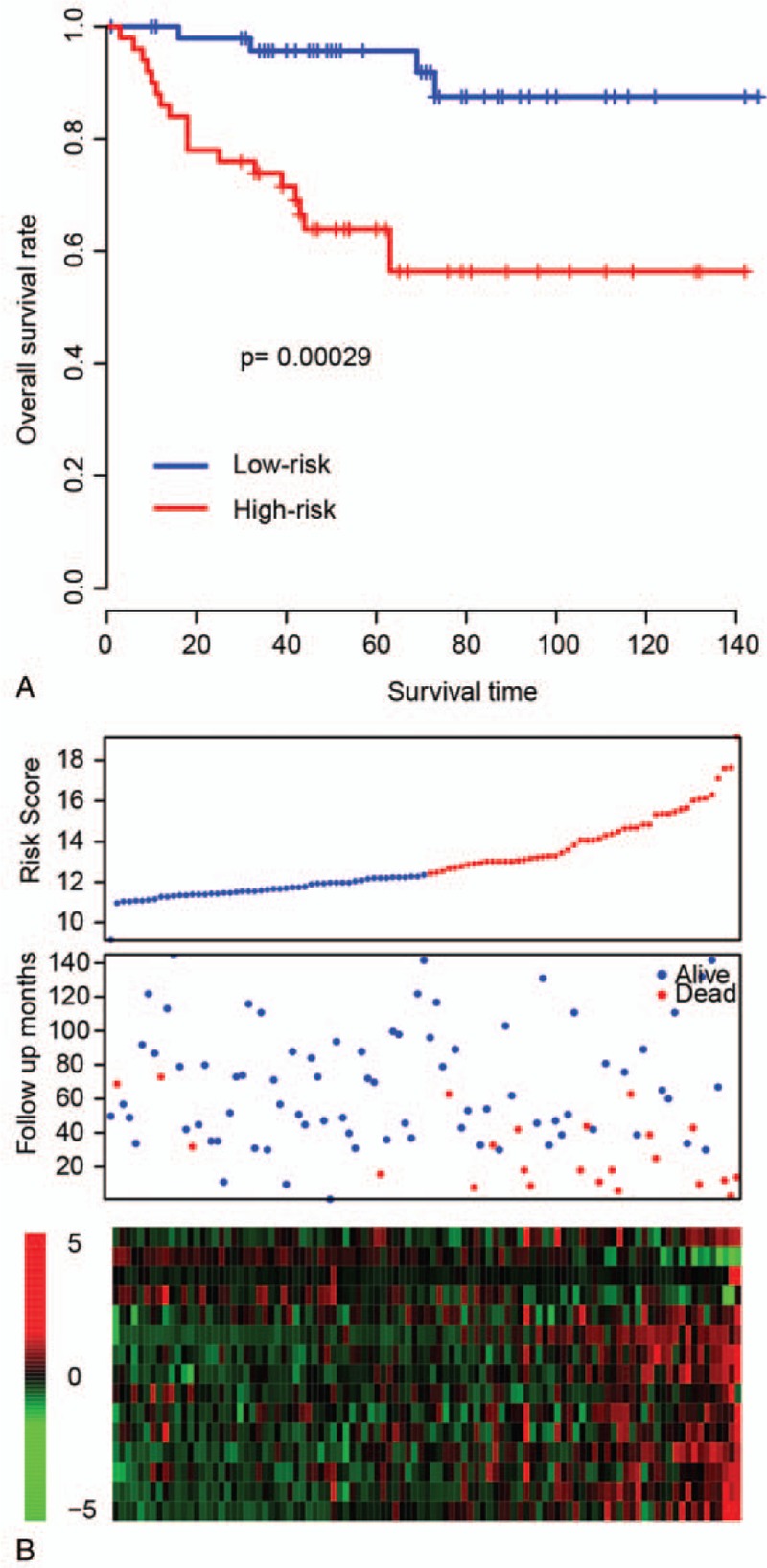
Risk score performance in other independent cohorts. (A) Survival differences for high-risk and low-risk groups in another independent dataset, E-MTAB-1980, resemble the profile of the training datasets, along with (B) gene expression.

The good performance of RS model may result from overfitness. To test our model, another dataset, E-MTAB-1980 (N = 101), which was generated from another platform (Aligent Microarray), was used for validation. The RS of each sample in E-MTAB-1980 dataset was calculated, and the samples were then divided into high-risk and low-risk groups according the median RS value of this dataset. Consistently with the result of TCGA dataset, the high-risk group in the E-MTAB-1980 dataset showed significantly worse survival than the low-risk group (*P* = .00029; Fig. 3A; Supplementary Table 2). The patients in the high-risk group had early events and relatively shorter OS. In addition, the gene expression pattern resembled the training dataset (Fig. 3B). All these results indicate that the RS model is valid across datasets and platforms.

### 3.4 Risk score and other clinicopathological indicators

**Figure 4:**
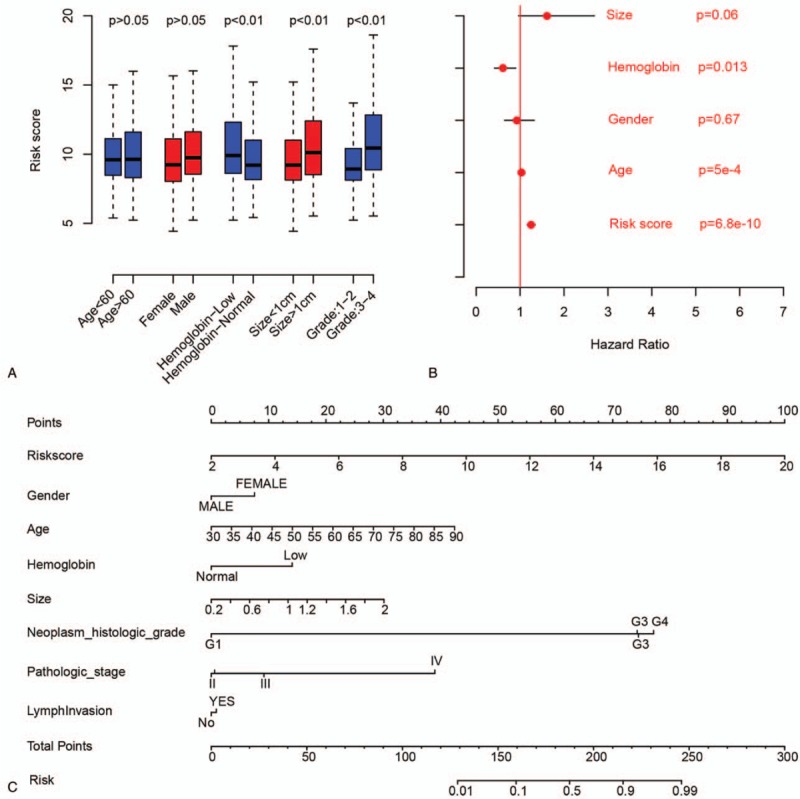
Risk score and other clinical indicators. (A) Box plot shows relationships between risk scores and other clinicopathological indicators. (B) Cox multivariate regression using clinical indicators and risk scores; red lines: 95% confidence interval; red dot: hazard ratio. (C) Three-year survival nomogram based on risk scores and other clinical indicators.

We investigated correlations between RS and other clinical indicators. The RS is independent of age and sex, but significantly associated with hemoglobin, primary tumor size, and grade (Fig. 4A). Cox multivariate regression showed that the RS was significantly associated with ccRCC prognosis (Fig. 4B), whereas other clinical indicators, including primary tumor size and sex, were not significantly associated with survival. A nomogram that considered RS, sex, hemoglobin, primary tumor size, histologic grade, pathologic stage, and lymph invasion was plotted for 3-year OS (Fig. 4C) in which RS had a wider range of risk points (0–100) than the other indicators. To assay the bias of the RS to clinical indicators, the samples were divided into subgroups according to clinical factors, including age (60 as cut-off), hemoglobin (normal or low), primary tumor size (1 cm as cut-off), pathological grade (1–2 or 3–4), stage (1–2 or 3–4), and lymph invasion. The prognostic value of RS was estimated in the subgroups, and showed that the RS is effective in all these subgroups (Fig. S1).

### 3.5 Risk score and radiation

**Figure 5:**
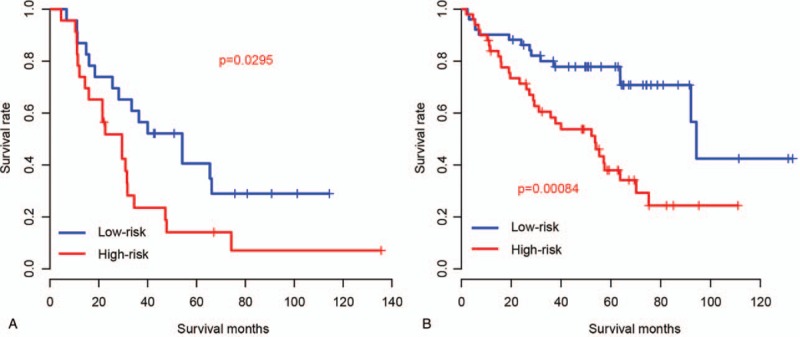
Risk score and radiation. Survival difference of high-risk and low-risk groups in radiation-depleted (A) and radiation-receiving (B) group was also significant.

Radiation is an important adjuvant therapy for ccRCC. To test whether the RS prognostic value was affected by radiation, TCGA samples were divided into radiation-receiving and radiation-depleted group (patients did not receive radiation), according to therapy records. Patients were divided into high-risk and low-risk groups. As expected, the high-risk group had a significantly worse survival than the low-risk group in both the radiation-depleted group (Fig. 5A) and radiation-receiving group (Fig. 5B), indicating that the prognostic value of RS was not affected by radiation.

## 4 Discussion

Outcomes of patients with ccRCC are determined by many factors, including surgery type, therapy methods, and genetic heterogeneity of ccRCC. Surgery and therapy methods are controllable, but genetic heterogeneity is not.^[2]^ Thus, single biomarkers often fail to predict survival across datasets, so a multiple biomarker-based model is needed. In this article, a RS model was developed and validated using gene expression, random forest variable hunting, and Cox regression. Subsequent analyses showed that the RS significantly indicated prognosis.

Among genes used for this model, CDC7, CDCA3, and ANAPC5 are involved in the cell cycle, and affect prognosis and migration in other cancer types.^[15–17]^ POFUT2 and ATP13A1 (enzymes), ADP ribosylation factor, GTPase activating protein 1, and collagen type VII alpha-1 chain were also included.

In the past years, multiple gene expression-based signatures have been developed to predict the progression of ccRCC.^[18]^ For example, combining expression of miR-21 and miR-126 led to good ccRCC survival prediction.^[19]^ A 5-gene expression-based model was developed using TCGA dataset, and another model combined clinical indicators and molecular biomarkers.^[20]^ However, these model lacks test datasets,^[21]^ and their samples were from single centers. Based on radiogenomics, a model was developed based on the molecular assay of ccRCC to predict survival.^[22]^ Rini et al^[23]^ reported that a 16-gene model was robust and effective in predicting recurrence after surgery for ccRCC. We assayed it in TCGA cohort, but could not validate it (not shown), as the model was developed and validated using Q-RTPCR platform. A CpG-methylation-based assay reportedly predicted survival in ccRCC (*P* = 1.4e-6) in TCGA cohort,^[24]^ but our model performed better (*P* = 5.6e-16). Our model was trained from a next-generation sequencing platform and was validated using microarray with a totally independent dataset. In conclusion, our model performed better.

This study had some limitations. This is a retrospective study, and may be inherently biased. Further prospective studies with more samples from different centers are needed to validate our findings.

## Acknowledgment

We thank Liwen Bianji, Edanz Group China (www.liwenbianji.cn/ac), for editing the English text of a draft of this manuscript.

## Author contributions

**Conceptualization:** Ping Li, He Ren, Zhaoli Zhou.

**Data curation:** Ping Li, Yan Zhang.

**Formal analysis:** Ping Li, Yan Zhang, Zhaoli Zhou.

**Investigation:** Ping Li, Yan Zhang.

**Methodology:** Ping Li, Yan Zhang.

**Project administration:** Ping Li, Yan Zhang.

**Resources:** He Ren, Yan Zhang.

**Software:** He Ren.

**Supervision:** He Ren.

**Validation:** He Ren.

**Visualization:** Ping Li, He Ren.

**Writing – original draft:** Ping Li, He Ren, Zhaoli Zhou.

**Writing – review & editing:** Ping Li, He Ren, Zhaoli Zhou.

## Supplementary Material

Supplemental Digital Content

## Supplementary Material

Supplemental Digital Content

## Supplementary Material

Supplemental Digital Content
