# Supplementary figures and images for: Fifteen-gene expression based model predicts the survival of clear cell renal cell carcinoma
Source: Medicine (Baltimore). 2018 Aug 17;97(33):e11839. doi: 10.1097/MD.0000000000011839 (PMC6113007; doi:10.1097/MD.0000000000011839)

Figure S1. Survival differences for high- and low-risk patients in different subgroups.


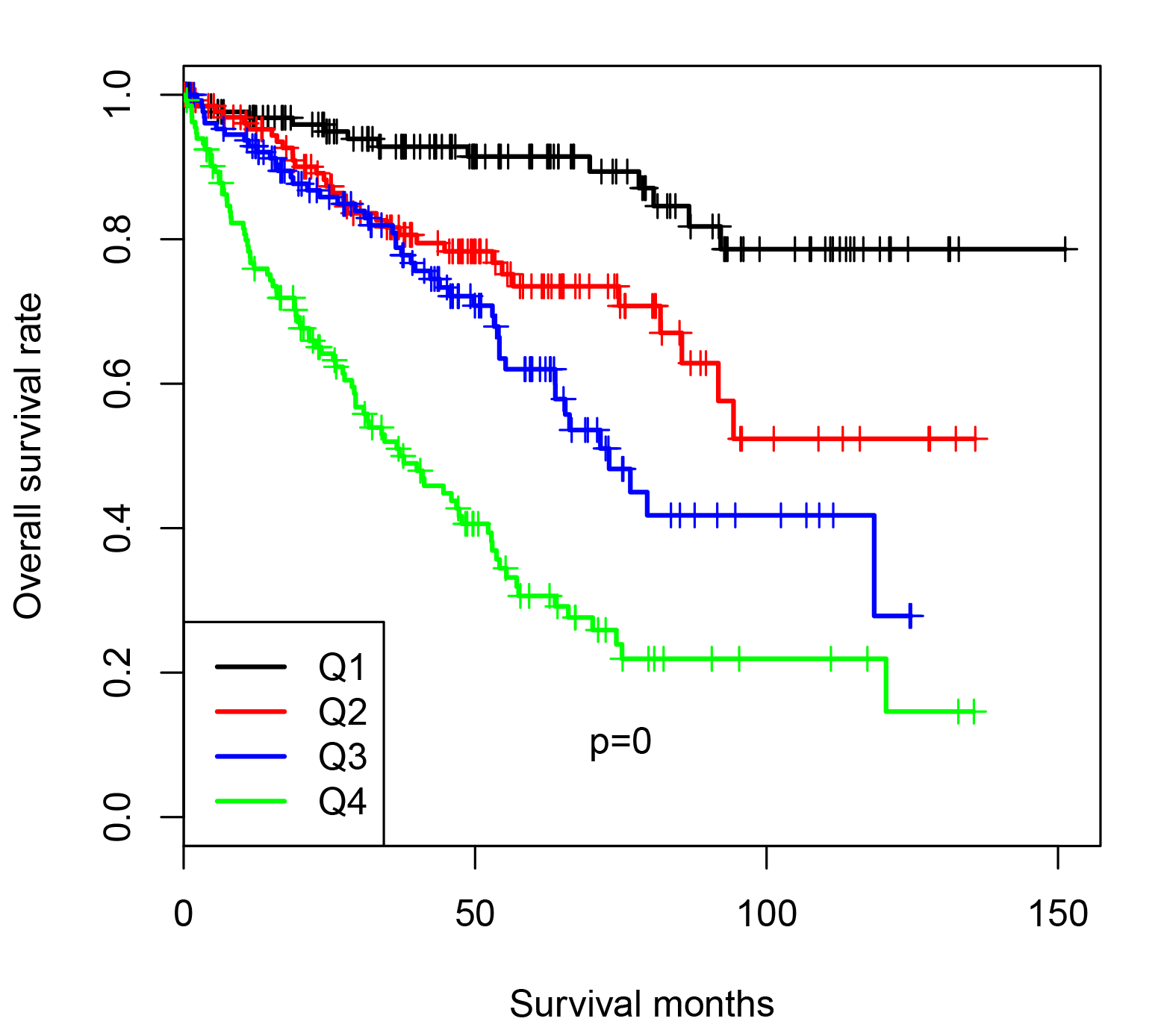

Supplement: Supplemental Digital Content [file medi-97-e11839-s001.doc]
